# Dynamic Artificial Neural Networks with Affective Systems

**DOI:** 10.1371/journal.pone.0080455

**Published:** 2013-11-26

**Authors:** Catherine D. Schuman, J. Douglas Birdwell

**Affiliations:** Department of Electrical Engineering and Computer Science, University of Tennessee, Knoxville, Tennessee, United States of America; McGill University, Canada

## Abstract

Artificial neural networks (ANNs) are processors that are trained to perform particular tasks. We couple a computational ANN with a simulated affective system in order to explore the interaction between the two. In particular, we design a simple affective system that adjusts the threshold values in the neurons of our ANN. The aim of this paper is to demonstrate that this simple affective system can control the firing rate of the ensemble of neurons in the ANN, as well as to explore the coupling between the affective system and the processes of long term potentiation (LTP) and long term depression (LTD), and the effect of the parameters of the affective system on its performance. We apply our networks with affective systems to a simple pole balancing example and briefly discuss the effect of affective systems on network performance.

## Introduction

Artificial neural networks (ANNs) are processors that are trained to perform a particular task, such as a classification, prediction or control task. One motivation of our research is to drive ANNs closer to their biological inspiration to explore the possible advantages of more flexible ANN structures and to gain insights into mechanisms of artificial, and perhaps natural, learning processes.

In our effort to move ANNs more towards biological neural networks, we propose the inclusion of a simple affective system in the ANN, so the ANN has two coupled parts: an affective system analogous to part of the limbic system and a computational system analogous to part of the cerebral cortex. This affective system communicates with both the computational system and the environment in order to achieve specified behavioral goals in the network (in this case, a desired firing rate). We demonstrate that this simple affective system can successfully control the firing rate of neurons in the network by changing the likelihood of the neurons to fire, which is analogous to releasing neurotransmitters in the system. We train our computational network with an affective system to complete a simple pole balancing task. The specific aims of this paper are to demonstrate that a network's firing rate can be adjusted using a simple affective system, and to demonstrate that the system can also readjust the firing rate level as needed. The mechanisms used to achieve these aims are tested using a large number of randomly generated ANNs, and both aggregate and statistical results are presented. Our primary interest is the exploration of interactions between a computational network and an affective system.

ANNs are designed to perform work analogous to functions typically implemented within the cerebral cortex. The cerebral cortex is responsible for the planning and execution of tasks in the human brain; it has distinct regions that carry out a variety of functions, including visual, auditory and memory-related functions [1]. ANNs, unlike the cerebral cortex, are trained to perform a highly specific, usually well-defined task. An ANN can be thought of as analogous to a very small network in the cerebral cortex. In the biological brain, the cerebral cortex receives information from the environment through sensory organs, and it interacts with the environment through, for example, neuromuscular stimulation. The operation of the cerebral cortex is influenced by activities in the lower parts of the brain such as those that play roles in short and long-term memory and affective (or emotional) subsystems. Our interest in this paper is the exploration of possible uses and benefits of affective/computational interactions in artificial neural systems.

An ANN, like the cerebral cortex, receives and transmits information to its environment, often through other mechanisms that play the roles of sensory systems. The interaction of the cerebral cortex with the basal ganglia and the limbic system, however, is typically not included in a neural network architecture. Among their many functions, parts of the limbic system are responsible for affective systems. Affective systems deal with emotions. Emotions are known to have an effect on the way we learn and reason [2]. Panksepp, in his work on affective neuroscience, defines four major emotional operating systems with their roots in the limbic system: seeking (curiosity), fear (providing, for example, a cautionary modulation of curiosity), panic (encouraging bonding among members of a population), and rage (defense or escape) [3]. Different parts of the limbic system are associated with different emotions. These affective systems communicate with the cerebral cortex through neurotransmitter pathways. These systems have an effect on learning; for example, a person with damage to the pathways that produce seeking behavior may be less curious, and thus, less likely to seek out new information about their environment. Biological affective systems are complex; the neurotransmitters associated with each system may differ and typically have different effects on the operation of the cerebral cortex and other regions within the central nervous systems, and the neurotransmitters may play different roles within different systems.

Variations of the concept of the seeking system have been presented in the machine learning literature. They are typically framed as reinforcement learning systems in which there is an internal (or intrinsic) reward for the agent exhibiting curiosity in its environment [4]–[15]. A variety of affective systems have also been modeled in the fields of human-computer interaction, human-robot interaction, and autonomous robots. These affective systems include fear, joy, sadness, and anger [16]–[20].

Emotional subsystems (such as seeking and fear) were evolved over time; that is, these systems emerged as useful and were propagated across generations. It is only recently that we have given these systems specific names and attributed to them the emotions that we feel. However, it is evident from biology that these systems are useful for learning [2], [3]. It is also evident from these works that a seeking type system in reinforcement learning problems improves performance on certain tasks [4]–[15].

These works from machine learning [4]–[20] have made use of artificial emotional systems where the emotions were predefined to evoke some sort of action in the client. Rather than attempt to create an artificial affective system analogous to known biological systems like seeking and fear, we instead propose evolving one or more affective systems alongside a computational network.

Based on the behavior of the biological affective system, the proposed affective systems emits “neurotransmitters” that change some component in the computational network. In particular, the demonstrated affective system changes the thresholds of neurons in the network, which is analogous to a neurotransmitter in the brain making neurons more or less likely to fire.

Some possible biological implications arose with our simulations. For example, we included long term potentiation (LTP) and long term depression (LTD) type processes in our simulations. When interacting with affective systems, these processes had interesting results, in some cases causing the behavior of the network to become unstable. Similar systems exist in biological systems, and either there is a mechanism present to ensure stability of behavior in the brain, or instabilities may also occur in the brain and may be related to abnormal behavior. These processes also had an interesting effect on the distribution of the synapse weight values. In particular, many of the synapses became very inhibitory, while few became very excitatory. In essence, the excitatory synaptic subnetwork became sparse relative to the entire artificial neural network. The fields of (biological) neuroscience and computational neuroscience may be synergistic, with opportunities for each to inform the other.

In the methods section of our paper, we introduce the features of our neural network, including affective systems. In the results section, we demonstrate that affective systems can control the firing rate of the network, and we include a brief example in which we co-evolve the computational system and affective system to complete a simple task. In the discussion section, we discuss possible biological implications of our results and propose further future directions for this work.

## Methods

The design of our artificial neural networks draws inspiration both from biological neural networks [1], [21], [22] and traditional artificial neural networks [23], [24] from machine learning. It is important to note that our goal is not to directly simulate a biological network, and our simulations are not intended to represent what occurs in the brain. Our model of a neuron is extremely simplified. We suspect that even with the relatively simple neural implementation we use we can generate complex behavior, by trading off complexity of the neuron for complexity in the network.

In our implementation, each neuron is located at a point in three-dimensional space. Neurons can be input neurons, output neurons, both types or neither type, depending on the requirements of the network. Each neuron has an associated threshold and refractory period. In preliminary implementations, both of these values are fixed for the network (i.e., every neuron in the network has the same threshold and refractory period). Neurons are connected to other neurons via synapses. These synapses are directed, so each neuron has a set of synapses *to* other neurons and a set of synapses *from* other neurons. The primary actions of a neuron are changes in charge and firing. Charge is received by a neuron from its synapses. The charge on a neuron is accumulated until that neuron's threshold is reached.

When the threshold is reached, if the neuron is *not* in its refractory period, the neuron fires, and the neuron's charge is reset to zero (or neutral, as the charge may also be negative). If the neuron is within its refractory period, then the neuron maintains its charge but does not fire. Thus, a neuron can accumulate charge during its refractory period, but it cannot fire during this period. As soon as a neuron fires, it enters its refractory period. This model of a neuron is inspired by the Hodgkin-Huxley model. In our present model, the charge values and threshold values of the neurons are bounded between -1 and 1.

Neuron thresholds and refractory periods, and synaptic propagation delays all introduce dynamic behaviors in the network. Unlike most proposed ANN architectures, but similar to natural neural processes, these dynamic effects are distributed throughout the network, and are directly influenced in our ANNs by the evolutionary programming methods we utilize to construct and adapt the ANNs for specific purposes.

Synapses in our implementation are defined by the neurons they connect. Specifically, each synapse goes *from* one neuron *to* another neuron. Each synapse has a distance between two neurons and a weight (or strength) of the synaptic connection. The distance between the two neurons affects how long it takes for charge to travel along the synapse. The weight of the synaptic connection determines how much charge arrives at the second neuron after the first neuron fires. Our network model does not currently include the concept of myelination; if two synapses are each of length 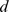, then it takes the same amount of time for charge to travel from one end of each synapse to the other.

The major actions associated with synapses are processes similar to long-term potentiation (LTP) and long-term depression (LTD). LTP and LTD occur in biological brains; it is speculated that they play a major role in learning [1]. If charge traveling along a synapse from neuron A to neuron B causes neuron B to fire, then the weight of that synapse increases. In our implementation, LTD occurs at that synapse if charge is received by neuron B during its refractory period. LTP increases the weight of the synaptic connection by a fixed value (specified for the network) and LTD decreases the weight of the synaptic connection by the same fixed value. Synapses have a refractory period associated with LTP and LTD, which prevents changes to the weights from occurring too rapidly.

It is important to note that, for most of the purposes of this paper, we could have omitted LTP and LTD altogether. However, the end goal of our work is to use an affective system to control or modulate the behavior of a neural network that is learning to perform a certain task. Learning for our networks causes not only the synapse weight values to change, but also the structure of the network to change. To demonstrate that an affective system can, in fact, control a learning network's behavior, we need to include some form of learning in the network. In our application to a particular task (a simple pole balancing example), learning is more complex in that the structure of the network also changes.

Our networks are defined as a grid in three-dimensional space. Maximum 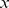, 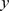, and 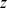 (called 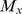, 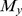, 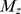) magnitudes are defined, as well as the granularity 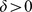 of the grid. Neurons may be located at coordinates in the grid, 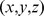, where 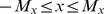, 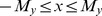, and 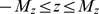, and the values of 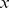, 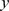, and 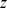 are integral multiples of the granularity 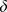. The granularity parameter specifies how close two neurons in the grid can be.

Simulations in our system take place at the network level and are discrete–event simulations. Networks have associated event queues, in which different event types are specified to occur at some time in the simulation. A unit of simulation time is the amount of time it takes for charge to travel one unit in space. For example, if two neurons are connected and are located one unit apart (i.e. a neuron at (0,0,0) and a neuron at (0,0,1)) then one unit of simulation time is the amount of time required for charge to travel from one of the neurons to the other.

Five event types are defined: addition of charge to a neuron, firing a neuron, adjustment of thresholds, an input pulse event, and a change in the desired firing rate. The addition of charge to a neuron and the firing of a neuron are internal events, which are caused by other events within the network. Input pulse events are events in which the network interacts with its environment. The adjustment of thresholds event is an interaction of the network with the simulated affective system. The change in the desired firing rate event is an interaction between the environment and the simulated affective system. Output events, in which the network gives information to the environment, can be defined for applications, such as the pole balancing application discussed in the results section.

The adjustment of thresholds event type applies a network-wide change to the threshold of every neuron in the network. The amount to change the threshold is determined by the affective system. The current firing rate of the network and the desired firing rate of the network are inputs to the affective system. The output of the affective system is the amount to change the thresholds by in the network.

Our affective system is determined by the following equations, which could be replaced by a second neural, or discrete-event, network. 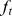 is the firing rate of the network, measured over a certain window, at time 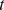. This is the input provided to the affective system from the network. 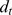 is the desired firing rate at time 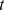. This desired firing rate is provided by the environment and can be changed by a desired firing rate event. The error at time 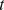, 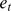, is calculated:
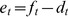
(1)


We have two affective systems: a simple affective system with two parameters and a slightly more complex affective system with three parameters. The simple affective system is used in all tests below, unless otherwise noted. Both affective systems have the parameter 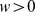, which is the window size of the system and specifies how often the error is recalculated. In the simple affective system, the change in the threshold at time 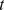 is calculated:
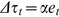
(2)


The parameter 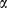 is a weighting term, and the change in the threshold at each time step is proportional to the firing rate error. 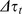 is the amount that every threshold in the network is changed at time 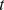. This result is passed back to the network, and the change is applied to *all* of the neurons in the network; since all of the neurons have the same initial threshold value of 0.5, all neurons in the network maintain the same threshold value throughout the simulation (except in the pole balancing task). The threshold is bounded to be in the interval 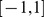 and equation (2) has no effect if it would cause either bound to be violated.

In the more complex affective system, a second parameter, 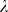, is added. A geometrically averaged error at time 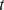, 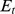 is calculated:
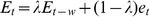
(3)


The parameter 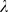 is a decay rate. It defines how much errors at times 0 through 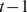 will affect the change in the threshold at time 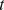. With this second affective system, the change in the threshold at time 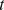 is calculated:
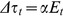
(4) where, again, 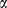 is a weighting term. In both cases, the result 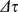 is passed back to the network, and the change is applied to all of the neurons in the network. Note that the first and second systems are equivalent if 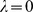. The same boundary logic applies as with equation (2).

A goal of this work is to demonstrate that a simple affective system interacting with an artificial neural network can have a noticeable effect and can stabilize the average firing rate at desired levels. All networks discussed in this paper (except for those trained to complete the pole balancing task) have 1000 neurons and 10000 synapses, where 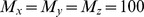. This is a relatively large artificial neural network, but compared to the human brain, this is a very small network. It is important to note, however, that we are not attempting to model a biological neural system with our artificial neural networks; our artificial neural networks are merely motivated by biology. The tasks these artificial networks are applied to are specific and well-defined. As such, they can be thought of as analogs to the small portions of the neocortex that implement specific functionalities. Networks with different numbers of neurons and synapses yield similar results, though they are not shown in this work.

The initial neuron placements in the network are random, and the distribution of the synapses is random, but with a higher likelihood of connectivity between spatially close neurons than neurons that are farther apart. In this network structure, there are 200 possible 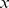-coordinate values, 200 possible 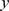 coordinate values and 200 possible 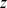 coordinate values, resulting in 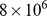 possible locations for neurons in the network. A specific instance or realization of a network has neurons at 1000 of these locations, randomly selected according to a uniform distribution, except no two neurons are allowed to occupy the same location.

Each network (except the networks trained to complete the pole balancing task) has a single input neuron that receives information from the environment. The “environment” in our setup consists of two things: pulses sent to the input neuron at exponentially-distributed random intervals, with a mean firing rate of 0.1 firings per unit time, and an input to the affective system that sets the current desired firing rate. This input plays the role of a persistent external excitation used to initiate and promote firing events in the network. This is an extremely simple environment; more complex tasks have richer environments that provide meaningful information to the network and receive signals produced by the network (see the pole balancing example below and [25]). The affective system monitors the behavior of the network and applies the threshold changes to the network every 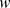 (the window size) units of simulation time. For all of the tests in this work, 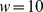.

All neurons in the network have a refractory period of one, which means that there is an upper limit on the firing rate of the network; since each neuron can fire at most once in a single simulated time step, the maximum firing rate of the network per time step is 1000. This assumes that the network is fully connected, which is not a requirement placed on the random initialization of the networks. There may be neurons that have no incoming synapses or neurons with no outgoing synapses, which would further limit the maximum firing rate of the network, and the network is not necessarily connected.

In our preliminary experiments, we set the parameters of the affective system to be 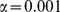 and 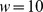. The long term potentiation/long term depression refractory periods are set to be 10, and the weights are adjusted up (for LTP) and down (for LTD) by 0.001. The parameters used in the pole balancing task are slightly different and are described in the Table S1.

## Results

### Single Desired Firing Rate

The first goal is to demonstrate that, even with these two very simple controlling affective systems, the network's firing rate can be adjusted and stabilized. The environment provides the single firing rate (a value between 50 and 950) to the affective system at the beginning of the simulation. The simulation is allowed to run for 10000 simulated units of time, where the input pulses are randomly applied as described above, and the affective system updates the threshold of the network every ten simulated time units (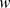 = 10).

**Figure 1:**
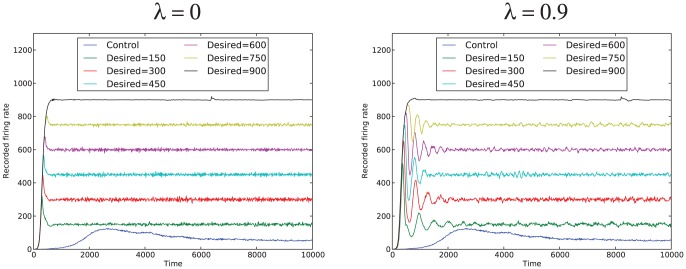
Simulation results for a single desired firing rate. Results for both 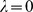 and 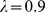 are shown.

We ran the same tests for 100 random network structures (each with 1000 neurons and 10000 synapses). Similar results were seen in each case. Figure 1 shows results for both the simple system (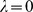) and the more complex system (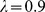), where 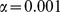, for one representative random network structure with 1000 neurons and 10000 synapses; the results are shown for a range of desired firing rates (150 to 900 in increments of 150). A control trace is also shown, demonstrating how the network behaves with the same set of input pulses, but without an associated affective system.

**Figure 2:**
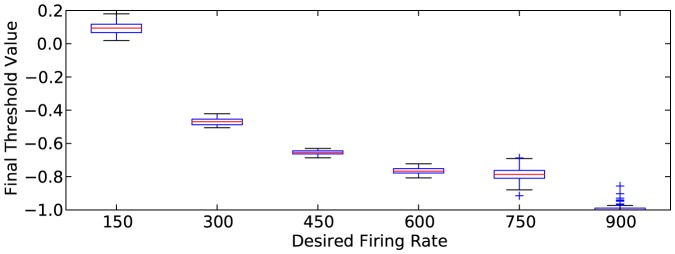
Box and whisker plots for the final threshold value. Plots shown for 100 networks at simulation completion for various desired firing rates. Results shown are for 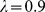; results for 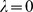 are nearly identical.

Figure 2 shows a box and whisker plot of the final threshold values after simulation has occurred, for the range of desired firing rates. The box and whisker plot shows these threshold values over the 100 random network structures. The resulting values of the thresholds are almost identical for both 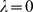 and 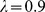, so only the results for 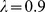 are shown. As expected, the resulting value of the threshold decreases as the desired firing rate increases. This is because neurons with low threshold values are more likely to fire than neurons with high threshold values. It is also important to note that when the neurons fire, their charge is set to neutral (0). This does not automatically force neurons with negative thresholds to fire. The neuron must receive some charge before it may fire again, and it must be outside of its refractory period to fire again.

As is evident from the graph, the simple affective system can successfully bring the firing rate close to the desired firing rate and maintain firing activity near the desired firing rate. However, there is oscillation around the desired firing rate in most cases, more so than is seen in the control case. The system with 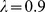 has more oscillation around the desired firing rate before it settles to the desired firing rate. In many cases this is undesirable behavior. However, as will be seen in later sections, for different values of 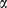, the complex system may be required in order for the system to stabilize.

### Multiple Desired Firing Rates

The second experiment seeks to demonstrate that not only can the controlling affective systems adjust the network firing rate to a particular level, but they can *readjust* the network firing rate level as needed over the course of simulation.

This test is important because the affective system is not the only regulatory system at work in our network architecture. LTP and LTD are also affecting the behavior of the network. Specifically, they are adjusting the weights in the network, and this adjustment is based on firing in the network. There is no guarantee that the new weight distribution will allow for the affective system to adjust the firing level. This test is to determine whether the affective system can continue to adjust the firing level despite the changes made by LTP and LTD.

In this experiment, the network continues to receive input pulses at exponentially distributed random intervals with a mean of 10, but the desired firing rate of the network is changed partway through the simulation, via a change in desired firing rate event sent by the environment and received by the affective system. In this section, results are shown for one network structure. Similar results occur for different network structures.

**Figure 3:**
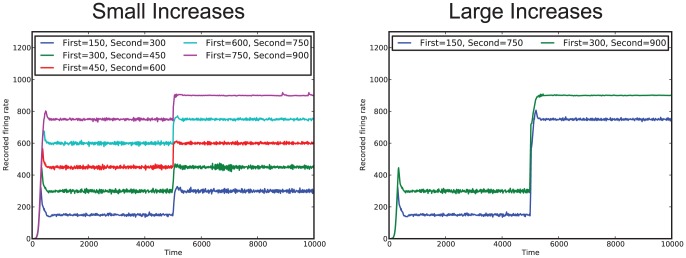
Simulation results for an increase in desired firing rates. Results for both small increases of 150 and large increases of 600 in the firing rate are shown.

**Figure 4:**
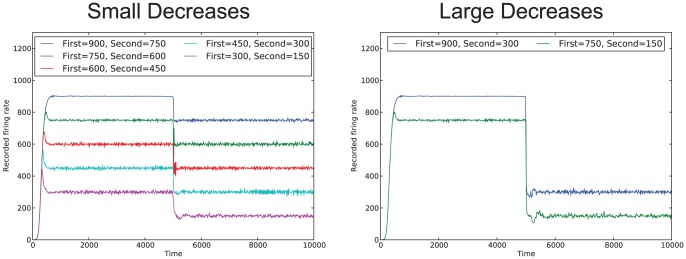
Simulation results for a decrease in desired firing rates. Results for both small decreases of 150 and large decreases of 600 in the firing rate are shown.

Figure 3 shows the simulation results for the simple system (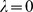) for cases where the desired firing rate of the system increases partway through the simulation; two different sizes of increases are shown (increases of 150 and 600). For both types of increases the affective system is able to adjust the thresholds to reach the first firing rate, and then readjust the thresholds to achieve the new firing rate, within a relatively short time period. Figure 4 show the simulation results for the simple system (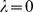) for cases where the desired firing rate of the system decreases partway through the simulation; two different sizes of decreases are shown (decreases of 150 and 600). Again, for both types of decreases the affective system is able to adjust the thresholds so that the network can achieve the original desired firing rate, and then readjust the thresholds to achieve the new firing rate. For all changes, there is a period of overcorrection of the affective subsystem; that is, for increases, the affective system adjusts the thresholds such that the firing rate usually ends up going higher than the desired firing rate (similarly for decreases). However, for smaller changes in the desired firing rate, there is less of an overcorrection by the affective system than there is for the higher firing rate. This is easily attributable to the design of the affective system; a larger change corresponds to a larger error, and thus a larger correction to the threshold value.

For the more complex affective system (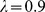), the results are similar, except, as in the single desired firing rate case, when adapting to a new desired firing rate, there is initially more oscillation around the desired firing rate. We have also seen, for these systems, that large decreases can cause unstable behavior (that is, the affective system cannot tune the thresholds to achieve the new desired firing rate).

**Figure 5:**
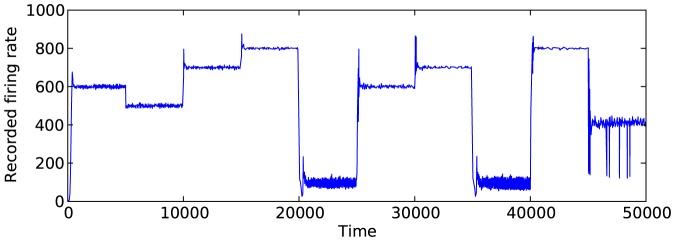
Simulation results for many changes in the firing rate. Results shown are for a network with 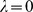.

Figure 5 shows a simulation over 50000 simulated time units in which the desired firing rate was adjusted several times for 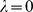. It demonstrates that the network can adjust to multiple firing rates over the course of simulation. However, there are some issues with instability when adjusting to later firing rates. These issues are related to LTP and LTD and are addressed in a later section.

### Affective System Parameters

Our current affective system has three parameters: 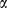 (a step-size parameter), 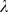 (a decay rate), and 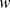 (the window size). For all experiments we have conducted thus far, we have used 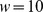. We chose this value because it averages out variations in firing rate in a small time period, but it also allows the affective system to update the thresholds frequently. Different window sizes may be considered in future work.

We define settling time as the time required for the network to achieve a desired firing rate and remain within 50 units of that desired firing rate. In the previous sections, we have shown how the results vary for two extreme values of 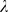 (0 and 0.9). In particular, we have found that for larger values of 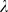, it takes longer for the network to settle at the desired firing rate, and there is more oscillation around the desired firing rate before the network settles. This behavior, however, is not consistent across a variety of desired firing rates; for instance, there is less oscillation at higher firing rates than there is for lower firing rates.

**Figure 6:**
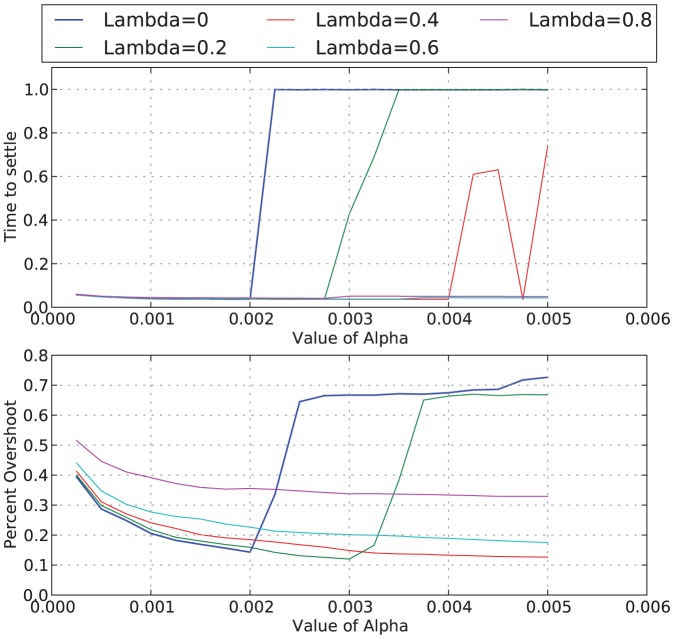
The effect of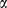 and 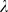 on time to settle and percent overshoot. Results shown are for one network structure and one desired firing rate.

Thus far, we have only presented results for a single value of 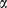 (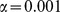). We chose this value of 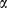 because the absolute maximum firing rate of the network is 1000, which means that the error between the desired firing rate and the current value of the network is at most (in the extreme case) 1000. When 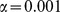 and 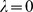, this bounds the change in thresholds to a magnitude of one. However, we also tried a variety of values of 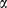, from 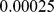 to 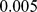 in increments of 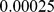. Figure 6 shows how the different values of 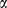 affect the percent overshoot of the firing rate (how high above the desired firing rate the recorded firing rate is, displayed as percent of the desired firing rate) and the time to settle (how long it takes the affective system to adjust the thresholds to achieve the desired firing rate).

**Figure 7:**
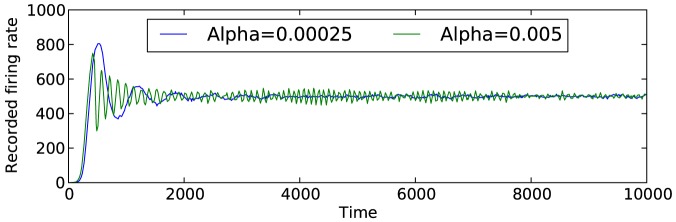
Simulation results for two extreme values of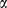. Results shown are for one network structure and one desired firing rate with 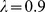. The two values of 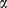 show are 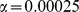 and 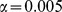.

Instability in this figure corresponds to a time to settle of 10000; in other words, settling is not achieved. This figure shows that, for higher values of 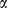, a high value of 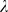 may be required to achieve stability. However, the percent overshoot increases as 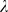 increases (except in the cases of instability). If 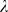 is positive, then 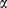 also affects the period of oscillation of the measured firing rate, prior to settling. Higher values of 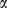 correspond to shorter periods of oscillations, as is shown in Figure 7. It is important to note that these figures only show the results for one network and one desired firing rate. Although the general conclusions still hold, the specific values of 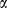 where instability becomes an issue differs for different networks.

### Structure of the Network

**Figure 8:**
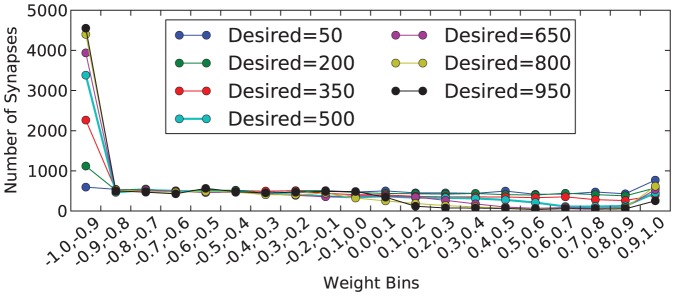
Histograms for the final synapse weights in the network at simulation completion. Results shown are for varying desired firing rates and one network structure with 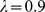.

Over the course of each simulation, the network topology is fixed; that is, the placement of neurons and synapses in the network is constant. However, the weights of the synapses change during the simulation due to the processes of LTP and LTD. Initially, for each network, the weights of the synapses are uniformly distributed between -1 and 1. In the following figures, we compute a histogram of the network weights across 20 bins. For a weight value of 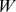, each bin is of the form 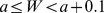, for 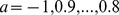, except for the last bin, which is 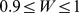. Figure 8 shows the histogram of the final weight values in the network, after the simulation is completed. This figure shows that, in general, most weights fall into either the 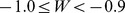 bin, with more weights falling into this bin as the desired firing rate increases. In all other bins up to the 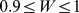, there are relatively few weights, and for smaller values of the desired firing rate, there is an increase in weights in the 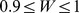 bin. This figure, along with Figure 2 indicates that, to achieve a higher firing rate, the threshold needs to be decreased, but the weights on the synapses also need to decrease, making most synapses in the network inhibitory synapses.

**Figure 9:**
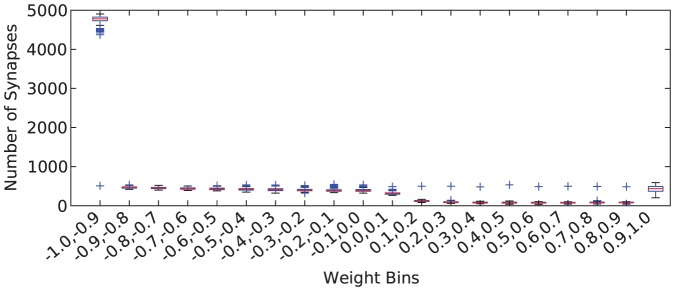
Box and whisker plots for the final synapse weights of 100 networks at simulation completion. Results shown are for desired firing rate of 900 and 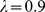.

These tests were run using 100 different randomly selected network structures. Figure 9 shows a box and whisker plot of the histograms of the weights in the final network structures for one desired firing rate (firing rate of 900). This figure shows that the results are consistent across these different network structures.

**Figure 10:**
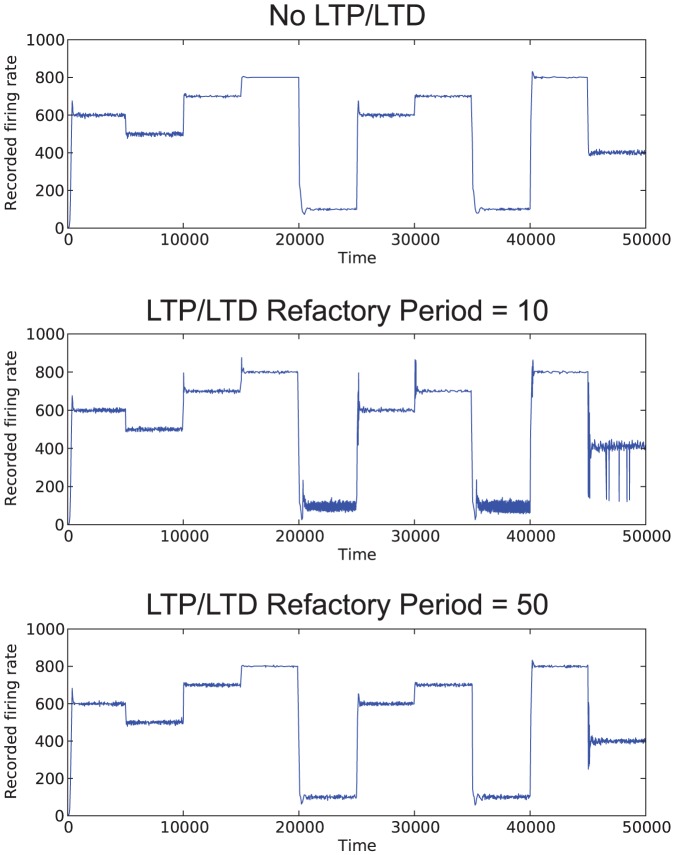
Simulation results for different LTP/LTD parameters. Results shown are for 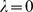.

As noted in the methods section of the paper, for the purposes of these simulations, LTP and LTD are not required. The affective system can tune the desired firing rate in the networks without these processes. However, we believe that, since LTP and LTD are expected to play an important role in day-to-day learning in the biological brain, it is prudent to include them in simulations of networks. The exclusion of LTP and LTD can lead to improved stability characteristics. Also, higher LTP and LTD refractory periods can result in lower instability. Figure 10 shows the simulation results for a network with no LTP/LTD, with LTP/LTD refractory period at 10 simulated time steps, and with LTP/LTD refractory period of 50 simulated time steps. As noted, the network without LTP/LTD was able to achieve its desired firing rates easily; however, the network with a higher LTP/LTD refractory period had similar results. This indicates that for these networks, learning (that is, any process that affects the structure of the network) should occur on a slower time scale than the affective system controller process.

**Figure 11:**
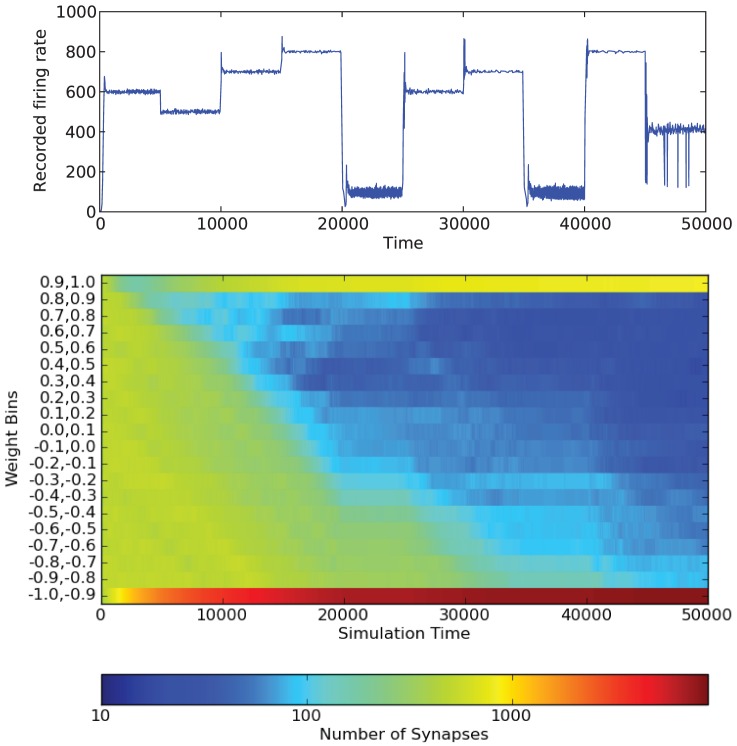
Color map showing the changes in synapse weights over a single simulation. Results shown are for 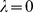. Note that the color bar is on a log scale. The simulation is also shown. The LTP/LTD refractory period used is 10.

**Figure 12:**
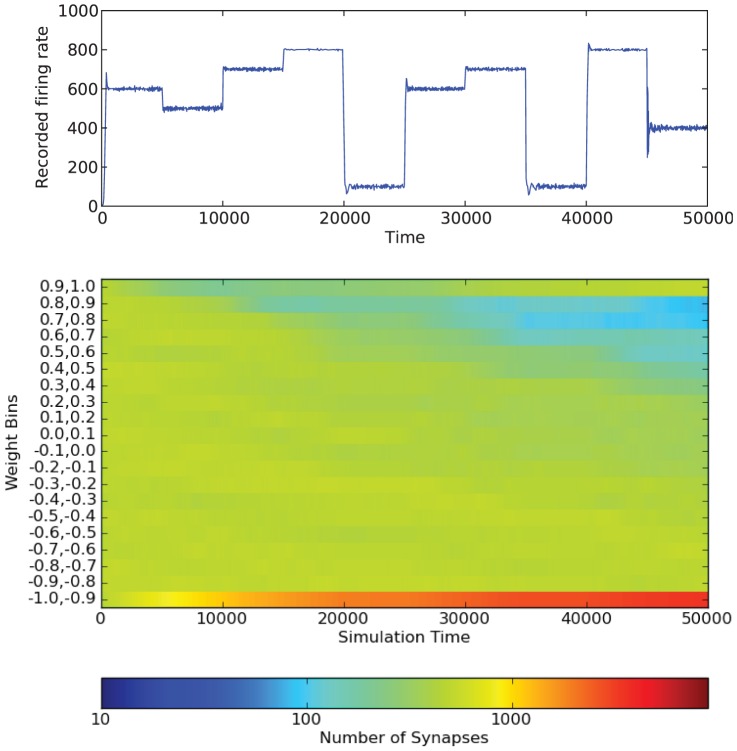
Color map showing the changes in synapse weights over a single simulation. Results shown are for 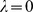. Note that the color bar is on a log scale. The simulation is also shown. The LTP/LTD refractory period used is 50.

Figure 11 shows how the weights change over the course of the simulation for one network structure and one desired firing rate. Similar results are obtained for different firing rates, but the rate at which the weights change is different for each desired firing rate. As expected by the results in Figure 8, the end result is that most weights fall into the 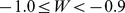 bin. As the simulation continues, more and more weights in the network fall into this bin. However, we also see an increase in the weights that fall into 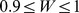 bin. This indicates that most weights in the network become either nearly fully inhibitory or excitatory. We can also see how different desired firing rates affect the synapse weights. For low firing rates (such as from 20000–25000 or 35000–40000), the weight values of the synapses stay mostly constant. Again, this is consistent with what we saw in Figure 8. Figure 12 shows how the weights changed over the course of the simulation when the LTP/LTD refractory period was set to 50 instead of 10. As expected, the weight values change much more slowly; however, the same basic trends occur.

### Application: Pole Balancing

We use an evolutionary algorithm as a training algorithm for our networks. A fitness function is defined for each application, and parents are selected using tournament selection [26]. Networks are represented and manipulated directly in this implementation. Both crossover and mutation operations are applied with some probability to the parents selected.. Details about the crossover and mutation operations as they apply to our network structures without affected systems are discussed in [25]. Both crossover and mutation operations are altered slightly to compensate for the inclusion of the simple affective systems. In particular, the desired firing rate is included as part of the training. In the crossover operation, the average desired firing rate of the parents is taken to be the desired firing rate in both children produced. A new mutation that sets the desired firing rate to a randomly selected value between zero and the number of neurons in the network is added to the mutation operation. The parameters of the evolutionary algorithm are given in the Table S2. We chose the parameters of the affective system for this application based on the results from previous sections. Those parameters are also given in the Table S1.

The pole balancing application is a widely used benchmark problem is both the machine learning [27], [28] and control engineering [29] fields. In this version of the pole balancing problem, a cart is assumed to be on a track so that it can move in only one dimension; that is, the cart can only be moved left or right. The track is assumed to be finite in that the cart must be kept between two points on the track. Attached to the cart is a pole. The pole is initially in some upright position. The goal of the pole balancing problem is to apply forces to the cart in order to keep the pole balanced and to keep the cart between the two endpoints of the track. In this work, we are using the bang-bang version of the problem, where there are only three possible actions: apply a force of -10 N, apply a force of 10 N, and apply no force at all to the cart. The pole balancing problem is discussed in detail in [30] and the equations and parameters used are included in Appendix S1 and Table S4.

The state of the pole balancing problem is described by four variables:

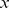
 = the position of the center of the cart on the track in meters.

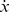
 = the velocity of the cart in meters/second.

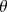
 = the angle of the pole from vertical in radians.

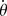
 = the angular velocity of the pole in radians/second.

In this work, a force is applied and the state will be updated every 0.02 seconds. The range of values for each state parameter is continuous. There are multiple ways to encode input for the system and decode output from the system. The output values for this problem are fairly straightforward, since there are three possible actions (apply a force of -10 N, apply a force of 10 N, and apply no force at all to the cart). In both examples, this is encoded using two output neurons. One corresponds to 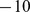 N and the other corresponds to 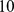 N. The output neuron that fires the most is the chosen action. If neither of the output neurons fires in the desired window, then no force is applied.

One option for encoding continuous valued input is to discretize the possible input space by splitting the space into ranges of values and having one input correspond to each range. To accomplish this, each of the four state space variables was split into three ranges, which are defined in Table S5. There is a corresponding input neuron for each of these ranges. When the state space is updated (or set initially), the range that each value belongs to is calculated, resulting in four ranges. Then, a pulse is applied to each of the four neurons. The network is then simulated for 200 units of simulated network time. At the end of these 200 time steps, the number of firings in that window for both output neurons is measured, and the action that corresponds to the output neuron that fired the most is applied. The state of the system 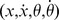 is then updated based on the force applied, and the process is repeated until a failure state is reached (the cart hits the end of the track or the pole falls) or the pole is kept up and the cart is kept between the tracks for some predefined time period.

The fitness function used in the evolutionary algorithm can have a major effect on the convergence rate of the system. For this problem, networks that are able to balance the pole longer should be maintained. That is, a straightforward fitness function for this problem is the amount of time the network can balance the pole. However, we would like for the system to be able to balance the pole from a variety of starting conditions (i.e. the pole in a various positions and the cart in various positions). The simplest possible fitness function would be to start in one state, measure how long the network is able to keep the pole balanced, and return that time as the fitness. However, this does not measure the network's ability to generalize. That is, a network that performs well starting from one state may perform poorly when starting from another state. To overcome this, initial state values can be used for each fitness test, and an average over their balancing times should be taken.

**Figure 13:**
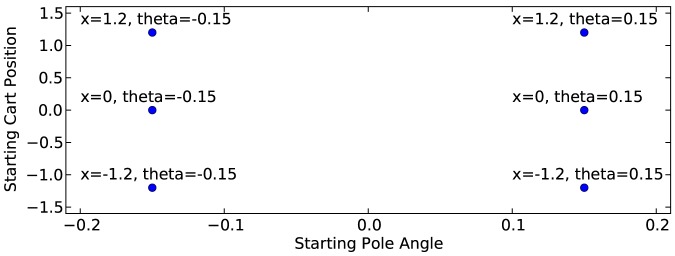
Six starting conditions for fitness function. This graph shows the six starting conditions that are tested as part of the fitness function for the simple pole balancing problem. 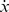 and 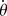 are assumed to be zero.

The evolutionary algorithm is trained until some pre-defined performance is achieved. We define a network as fully-trained in the pole balancing problem if that network can keep the pole balanced and the cart between the endpoints for 300 seconds for each of the six test cases shown in Figure 13, where the linear and angular velocities are zero.

Using this measure of performance, sixteen networks were evolved that satisfy the performance requirement. As part of training, the desired firing level was also found. We also obtained networks using the same random seeds (and thus the same initial populations) as the networks with the affective systems, and trained them without affective systems. Using these sixteen random seeds, only twelve of the networks converged in 100 epochs (whereas the average convergence time in epochs for networks with affective systems was 33.88). Since the other four did not converge in 100 epochs, they are not included in the results given below. It is worth noting that the results presented here are not conclusive because the number of networks found at this point is relatively small. Future work may expand upon the results shown here.

**Figure 14:**
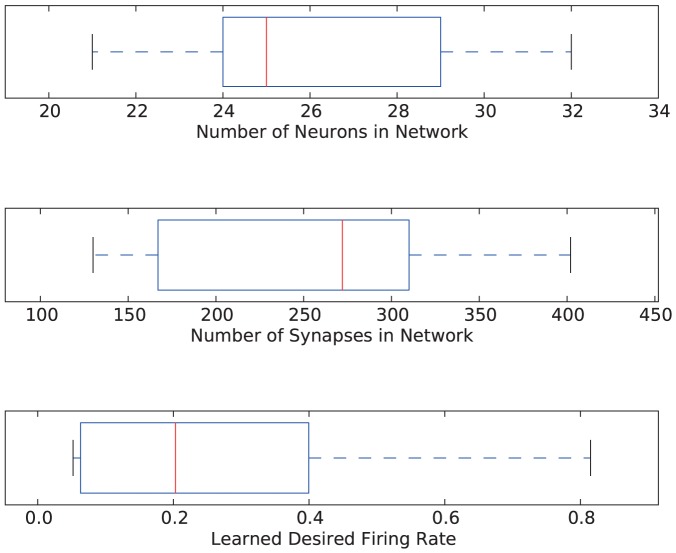
Box and whisker plots summarizing the complexity of the networks. The number of neurons, the number of synapses and the desired firing rate are shown for the sixteen networks trained with affective systems.

Box plots showing the sizes of the networks trained with affective systems (in terms of neurons and synapses), as well as the resulting desired firing rates for those networks are given in Figure 14. The average number of neurons in a network trained with an affective system is 24.83, while the average number of neurons in a network trained without an affective system is 25.25. Similarly, the average number of synapses in a network trained with an affective system is 223, while it is 239.25 in a network trained without an affective system. We do not view these differences as representative because of the small sample size. However, we expect the networks with affective systems to be smaller than networks without affective systems, because of the extra level of complexity in operation that the affective systems add.

The resulting networks were tested to determine how well they generalized their solutions to the pole balancing task. To determine this, 10 random starting conditions were chosen from each of the following sets of ranges: 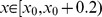, 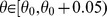, where 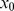 started as 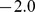 and continued to 1.8 in increment of 0.2, and 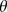 started at −0.15 and continued to 0.1 in increments of 0.05, resulting in a total of 1260 total test runs. On the contour plots, the points shown are the averages of the 10 random starting conditions in 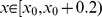, 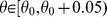.

We ran this test on three different network types: the networks trained with the affective systems; the same networks trained with the affective system, but not including the affective system; and networks trained without the affective system.

**Figure 15:**
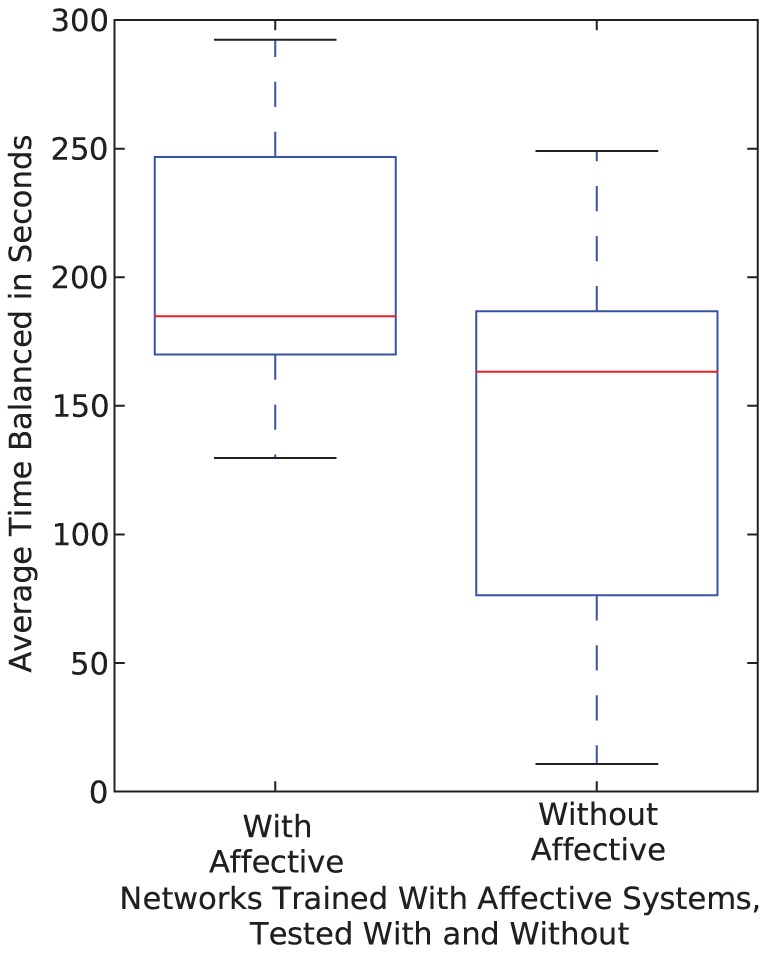
Box and whisker plots showing the results for networks tested with and without affective systems. The results shown in the box plots are the overall averages of the sixteen networks over 1260 test cases (10 random points chosen in each of 126 boxes).

Figure 15 shows the results when the sixteen networks that were trained with the affective system underwent the generalization tests with and without the affective system operating during the tests. As can be seen in this figure, the operation of the affective system has an effect on the performance of the system. In particular, the networks with the affective system generalized their solutions better than the networks without affective systems. This is to be expected, because the networks were trained to operate with the affective system. However, we saw two types of networks emerge in that group of sixteen: one group in which the affective system was vital for good performance and one group in which the affective system was non-vital. The affective system was absolutely required for more than half of the sixteen networks in order to have good performance. It is likely that the inclusion of the affective system in training but not in operation helped find better thresholds.

**Figure 16:**
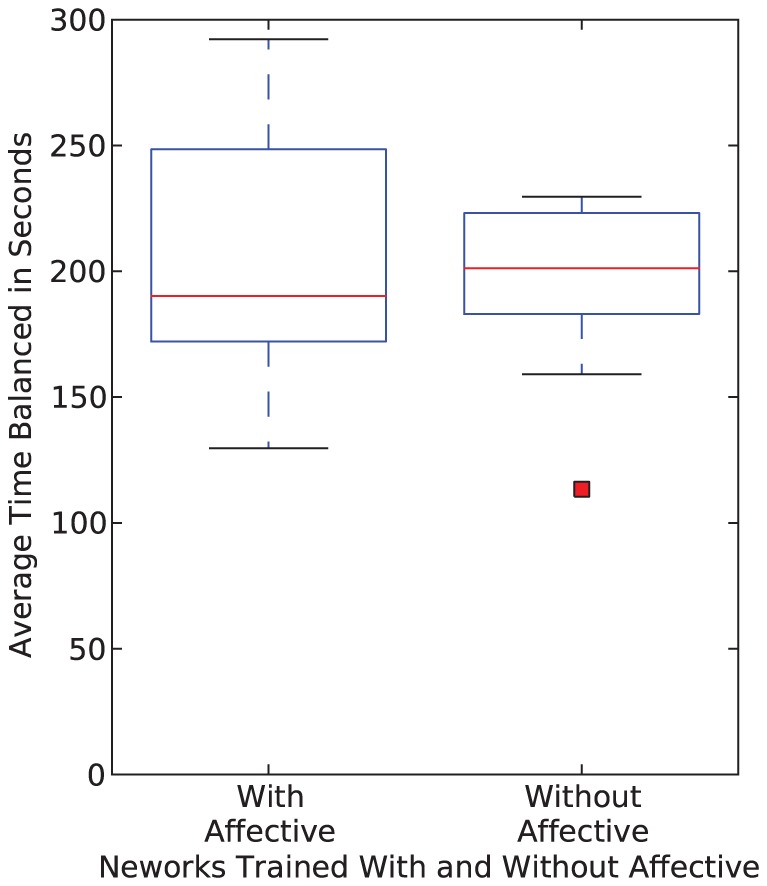
Box and whisker plots showing the results for networks trained with and without affective systems. The results shown in the box plots are the overall average of the networks over 1260 test cases. Since only twelve of the networks trained without affective networks converged, the results are shown only for those twelve networks, as well as the twelve networks with affective systems with the corresponding random seeds.

Figure 16 shows the results of the twelve networks trained without affective systems (of the 16 that satisfied the criterion for completion of training), when compared with the same seeded twelve networks trained with affective systems. Again, it appears as though the networks trained with the affective systems perform better than those trained without affective systems. However, the difference is relatively small, especially when considering the small sample size. Also, only 75 percent of the training tasks completed without the affective system, whereas 100 percent of the training tasks that incorporated the affective subsystems completed.

**Figure 17:**
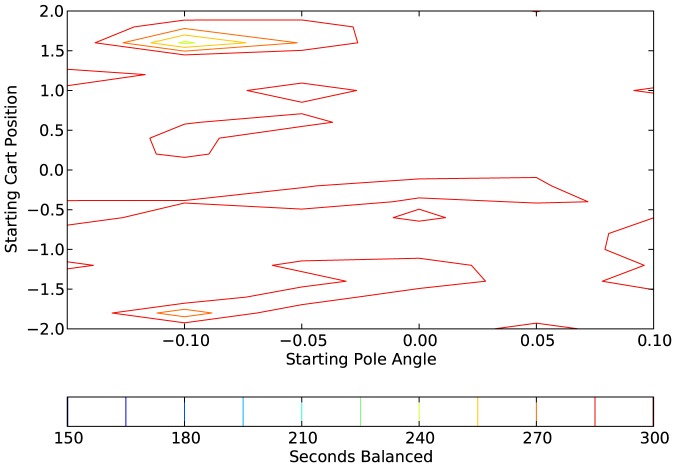
Plot showing the generalization results on a network with an affective system. Each point on the contour plot is the average of 10 test cases.

**Figure 18:**
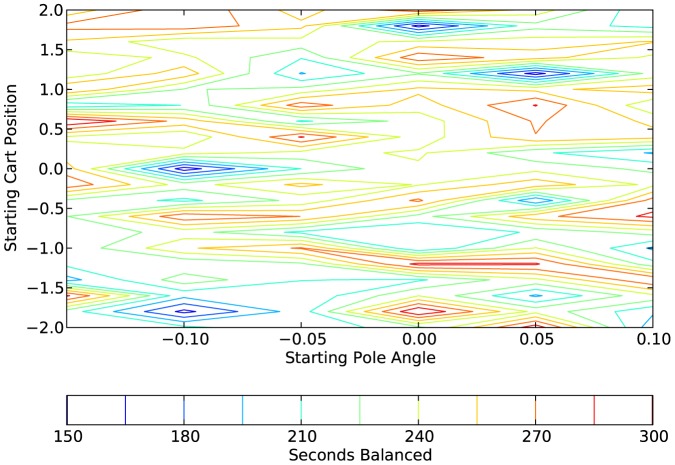
Plot showing the generalization results on a network tested without an affective system. The network in this case is the same network as in Figure 17, but the affective system is not operating during the tests. Each point on the contour plot is the average of 10 test cases.

**Figure 19:**
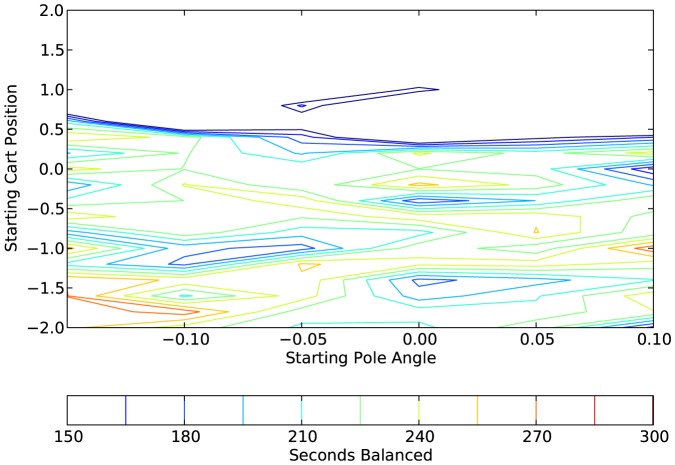
Plot showing the generalization results on a network trained without an affective system. The network was trained without an affective system, but the original population on the evolutionary algorithm was the same as the population for Figure 17 (they used the same random seed). Each point on the contour plot is the average of 10 test cases.

Figures 17, 18, and 19 show, respectively, the results for one network that was trained with the affective system and tested with the affective system, the same network tested without the affective system, and a network that was trained without the affective system, but started with the same initial evolutionary algorithm population. Each point on the contour map is the average of the 10 runs that correspond to that range. For this example, the network with the affective system performed very well on the generalization test. The network trained with the affective system but tested without it performed second best; however, it is clear that the affective system was vital for the good performance of this network. Finally, the network trained without the affective system performed worst. In this case, the inclusion of the affective system in training vastly improved the generalization performance of the network.

In general, we are heartened by the results given here. Though many more tests are required to form a conclusive statement about the effectiveness of networks with affective systems trained to complete tasks, we certainly believe that it is worth exploring further.

## Discussion

This work has established that a simple affective system can control an aspect of the behavior of a computational system (specifically, the firing rate of that computational system). We established the system's ability to achieve and maintain a desired firing rate, as well as to adapt to changes in the desired firing rate. We also saw the effect of the inclusion of an affective system when training to complete a specific task.

We noted some of the effects that processes such as LTP and LTD can cause in this type of system. LTP and LTD are known to enable cognitive-like characteristics in ANNs such as associative memory and classification [31], [32]. The simplified artificial system may provide some insight into the behavior of the biological brain. We have shown that LTP and LTD can have drastic effects on the behavior of the system. Specifically, if the affective system is not behaving correctly with respect to the system (i.e. the wrong parameters are being used), then these processes may cause the behavior of the network to become unstable (as shown in Figure 5).

We also saw that when LTP and LTD occurred at the same rate as interactions with the affective system occurred, the behavior of the network was sometimes unstable. On the other hand, when LTP and LTD occurred at a slower rate than the interactions with the affective system, there was less instability in the system (as shown in Figure 10). This indicates that the interactions between multiple subsystems can be controlled to some extent through separation of time scales, which corresponds to the ranges of the parameter values in these computational experiments.

The processes of LTP and LTD are the only processes by which synapse weight values are changed in our simulation (except in the pole balancing example). As shown in Figure 8 and 12, these processes have a major effect on the way that weight values change. Specifically, we see that many of the synapses become very inhibitory over the course of simulation, and there is also a subset that become very excitatory. In particular, these excitatory synapses are the ones that contribute primarily to the firing in the system. So, there are particular excitatory pathways that are emerging that stimulate firing in the system. The rate at which the synapse weight values change is also related to the firing rate of the system. We speculate that this will be important in our future work, because higher firing rates may correspond to more productive systems.

As noted in the introduction, it is known that affective systems impact the way that humans learn and reason. In [3], four major affective operating systems are defined: seeking, fear, panic, and rage. It is easy to see that the inclusion of one of these systems may be helpful for learning a particular task. Seeking, for example, would be useful in a task that requires exploration of an environment. Fear would be useful in a task predator-prey environment. Panic, a system that deals with social-bonding, would be helpful in a task where multiple agents must learn to work as a team. Rage would be helpful in a task that requires protection or defense of an object. The missing piece, however, is how to model one of these systems for a particular network and a particular task. It is difficult to simulate these systems; moreover, it may even be difficult to establish which of the four may be appropriate for any given task.

The important insight in observing biological affective systems is that these systems were not designed *a priori* to complete a certain task. These systems evolved over time to become the seeking, fear, panic and rage systems biologists can identify today. They have utility in not only humans, but in many other biological organisms through evolutionary processes, and they propagated to future generations. Rather than designing, for example, an affective system that makes a computational network “curious,” it makes more sense to evolve an affective system that leads to improved performance for a particular task in a particular environment.

The training scheme of the computational portion of our ANN is an evolutionary algorithm [25]. We showed how the inclusion of our simple affective system affects training and performance on a simple task. The affective system is simple, but more complex systems could be used. The current results provide support for the utility of a combined computational/affective systems approach in machine learning. Instead of using these simple equations, we could use a network similar to that of the computational network. The affective system can be implemented using equations or a network and receives information from the environment and the computational network. Its outputs, as above, communicate how the thresholds in the computational network should be changed. Then, the affective system could be trained using a similar (or identical) evolutionary algorithm as the computational system. Using this learning scheme, the networks can be co-evolved to solve a task. In this way, the overall system develops whatever type of affective system is required to solve a certain task. Moreover, the affective system will no longer be a static component that affects the network in exactly the same way, regardless of inputs, but instead will take into account the current state of the environment in order to determine how to affect the computational network.

This affective system is also largely simplified in the way it affects the computational network; in particular, the thresholds in the computational network are all changed uniformly. A second step in developing a more complex affective system would be to allow the affective system to control which part of the network is affected by the changes in threshold, and to allow for non-uniform changes.

## Supporting Information

Table S1 **Network and Affective System Parameters.**
(PDF)

Table S2 **Evolutionary Algorithm Parameters.**
(PDF)

Table S3 **Mutation Types and Rates.**
(PDF)

Table S4 **Pole Balancing Parameters.**
(PDF)

Table S5 **Input Encoding Ranges**. Each of the four state space variables were split into three ranges. As described in the results section, there is a corresponding input neuron for each of these ranges. When the state space is updated (or set initially), the range that each value belongs to is calculated, resulting in four ranges. Then, a pulse is applied to each of the four neurons.
(PDF)

Appendix S1 **Details and equations used in pole balancing example.**
(PDF)
