# Supplementary material for: Dynamic Artificial Neural Networks with Affective Systems
Source: PLoS One. 2013 Nov 26;8(11):e80455. doi: 10.1371/journal.pone.0080455 (PMC3841186; doi:10.1371/journal.pone.0080455)
Supplement: Table S1 — Network and Affective System Parameters. (PDF) [file pone.0080455.s001.pdf]

Table S1: **Network and Affective System Parameters**

**Parameter**	**Value**
M_x	100
M_y	100
M_z	100
Network granularity δ	1
α	0.001
λ	0
LTP/LTD refractory	100 steps of simulated time
Amount LTP/LTD adjusted	0.001
Window size w	20
