# Supplementary material for: Dynamic Artificial Neural Networks with Affective Systems
Source: PLoS One. 2013 Nov 26;8(11):e80455. doi: 10.1371/journal.pone.0080455 (PMC3841186; doi:10.1371/journal.pone.0080455)
Supplement: Table S2 — Evolutionary Algorithm Parameters. (PDF) [file pone.0080455.s002.pdf]

Table S2: **Evolutionary Algorithm Parameters**

**Parameter**	**Value**
Population size	500
Mutation rate	0.9
Crossover rate	0.9
Tournament size	20
