# Supplementary material for: Dynamic Artificial Neural Networks with Affective Systems
Source: PLoS One. 2013 Nov 26;8(11):e80455. doi: 10.1371/journal.pone.0080455 (PMC3841186; doi:10.1371/journal.pone.0080455)
Supplement: Table S3 — Mutation Types and Rates. (PDF) [file pone.0080455.s003.pdf]

Table S3: **Mutation Types and Rates**

**Mutation Type**	**Mutation Rate**
Change sign of the weight of a randomly selected (RS) synapse	0.267
Randomly change the weight of a RS synapse	0.267
Add a synapse between two RS neurons that are not already connected	0.133
Delete a RS selected synapse	0.133
Add a neuron at a RS position	0.027
Delete a RS neuron	0.013
Change the threshold of a RS neuron	0.027
Change the desired firing rate to a RS value between 0 and the number of neurons	0.133
