# Supplementary material for: Dynamic Artificial Neural Networks with Affective Systems
Source: PLoS One. 2013 Nov 26;8(11):e80455. doi: 10.1371/journal.pone.0080455 (PMC3841186; doi:10.1371/journal.pone.0080455)
Supplement: Table S4 — Pole Balancing Parameters. (PDF) [file pone.0080455.s004.pdf]

Table S4: **Pole Balancing Parameters**

**Parameter**	**Value**
Cart's mass (m_c)	1 kg
Pole's mass (m_p)	0.1 kg
Cart length	1 m
Length of pole (l)	1 m
Track length	5.8 m
τ	0.02 sec
