# Supplementary material for: Dynamic Artificial Neural Networks with Affective Systems
Source: PLoS One. 2013 Nov 26;8(11):e80455. doi: 10.1371/journal.pone.0080455 (PMC3841186; doi:10.1371/journal.pone.0080455)
Supplement: Table S5 — Input Encoding Ranges. Each of the four state space variables were split into three ranges. As described in the results section, there is a corresponding input neuron for each of these ranges. When the state space is updated (or set initially), the range that each value belongs to is calculated, resulting in four ranges. Then, a pulse is applied to each of the four neurons. (PDF) [file pone.0080455.s005.pdf]

Table S5: **Input Encoding Ranges**. Each of the four state space variables were split into three ranges. . As described in the results section, there is a corresponding input neuron for each of these ranges. When the state space is updated (or set initially), the range that each value belongs to is calculated, resulting in four ranges. Then, a pulse is applied to each of the four neurons.

**Parameter**	**Ranges for Encoding**
x	$(-\infty, -0.8], (-0.8, 0.8], (0.8, \infty)$
$\dot{x}$	$(-\infty, -0.66], (-0.66, 0.66], (0.66, \infty)$
θ	$(-\infty, -0.0697], (-0.0697, 0.0697], [0.0697, \infty)$
$\dot{\theta}$	$(\infty, -0.667], (-0.667, 0.667], (0.667, \infty)$
