# Supplementary material for: Dynamic Artificial Neural Networks with Affective Systems
Source: PLoS One. 2013 Nov 26;8(11):e80455. doi: 10.1371/journal.pone.0080455 (PMC3841186; doi:10.1371/journal.pone.0080455)
Supplement: Appendix S1 — Details and equations used in pole balancing example. (PDF) [file pone.0080455.s006.pdf]

Appendix S1

The track ranges from -2.9 meters to 2.9 meters, so the minimum and maximum are selected as -2.4 and 2.4. The two actions are $-10N$ and $10N$. In this work, a force will be applied and the state will be updated every 0.02 seconds.

Given x, $\dot{x}$, θ, $\dot{\theta}$ and the force applied, the new state is determined using the following model. If $\dot{\theta} = \omega$, the following equation ([1]) can be used to determine the angular acceleration, where F is the force, g is the gravitational constant ($9.8m/sec^2$):

$$\dot{\omega} = \frac{m_c g \sin(\theta) - \cos(\theta)[F + m_p l \dot{\theta}^2 \sin(\theta)]}{(4/3)m_c l - m_p l \cos(\theta)^2} \tag{1}$$

Then, if $\dot{x} = v$, the acceleration of the cart can be determined with the following equation ([1]):

$$\dot{v} = \frac{F + m_p l[\dot{\theta}^2 \sin(\theta) - \dot{\omega}\cos(\theta)]}{m_c} \tag{2}$$

The dynamic behavior of the cart and pole system is approximated using Euler's first-order numerical integration rule. Using this rule, the new cart position can be approximated using the following equation, where $\tau = 0.02$:

$$x(t+\tau) = x(t) + \tau\dot{x}(t) \tag{3}$$

In the same manner, the new angle of the pole can be determined with the following equation:

$$\theta(t+\tau) = \theta(t) + \tau\dot{\theta}(t) \tag{4}$$

Then, the new velocity of the cart, $v = \dot{x}$, can be determined with the following equation:

$$\dot{x}(t+\tau) = v(t+\tau) = v(t) + \tau\dot{v}(t) \tag{5}$$

The new angular velocity of the cart, $\omega = \dot{\theta}$, can be determined with the following equation:

$$\dot{\theta}(t+\tau) = \omega(t+\tau) = \omega(t) + \tau\dot{\omega}(t) \tag{6}$$

The networks fails under two conditions: (1) the cart hits the end of the track ($x >= 2.4$ m or $x <= -2.4$ m) or (2) the pole falls ($\theta >= 0.209$ radians or $\theta <= -0.209$ radians).

References

1. Anderson C (1989) Learning to control an inverted pendulum using neural networks. Control Systems Magazine, IEEE 9: 31 -37. 1
